# REM versus Non-REM sleep disturbance specifically affects inter-specific emotion processing in family dogs (*Canis familiaris*)

**DOI:** 10.1038/s41598-020-67092-5

**Published:** 2020-06-26

**Authors:** Henrietta Bolló, Krisztina Kovács, Radu Lefter, Ferenc Gombos, Enikő Kubinyi, József Topál, Anna Kis

**Affiliations:** 1 Doctoral School of Psychology, ELTE Eötvös Loránd University, Budapest, Hungary; 2 Institute of Psychology, ELTE Eötvös Loránd University, Budapest, Hungary; 3 Institute of Cognitive Neuroscience and Psychology, Research Centre for Natural Sciences, Budapest, Hungary; 4 Alexandru Ioan Cuza University, Iasi, Romania; 5 Pázmány Péter Catholic University, Budapest, Hungary; 6 ELTE Eötvös Loránd University, Budapest, Hungary; 7 MTA-PPKE Adolescent Development Research Group, Budapest, Hungary; 8 Department of Ethology ELTE Eötvös Loránd Universi, Budapest, Hungary

**Keywords:** Cognitive neuroscience, Psychology

## Abstract

Dogs have outstanding capabilities to read human emotional expressions, both vocal and facial. It has also been shown that positively versus negatively valenced dog-human social interactions substantially affect dogs’ subsequent sleep. In the present study, we manipulated dogs’ (N = 15, in a within subject design) sleep structure by specifically disrupting REM versus Non-REM sleep, while maintaining equal sleep efficiency (monitored via non-invasive polysomnography). We found that both the number of awakenings as well as relative Non-REM (but not relative REM) duration influenced dogs’ viewing patterns in a task where sad and happy human faces were simultaneously projected with sad or happy human voice playbacks. In accordance with the emotion laterality hypothesis, the interaction between sound valence and Non-REM sleep duration was specific to images projected to the left (regardless of image-sound congruency). These results reveal the first evidence of a causal link between sleep structure and inter-specific emotion-processing in the family dog.

## Introduction

Sleep is crucial for normal emotional functioning^1^. The finding that nearly all psychiatric disorders express co-occurring sleep abnormalities^2^ clearly illustrates the relationship between sleep and emotions. Sleep deprivation paradigms have been used in the case of humans and also non-human animals to approve the causal link between disturbed sleep and impaired emotion-related behaviours.

Sleep deprivation causes a decrease in emotion recognition performance in humans^3,4^. For example response latencies increase while accuracy decreases in an emotional face recognition task after sleep deprivation, compared to a non-sleep-deprived condition^5^. Furthermore, such effects are more pronounced when the stimulus is in the left visual field, suggesting that the right hemisphere is more severely impacted by sleep deprivation, compared to the left.

In parallel to the above research, evidence has been accumulated to support the *Right Hemisphere Model* of emotion processing^6^, which suggests that the right hemisphere is dominant for decoding emotional information, regardless of its valence. However, there is another theory on lateralized emotional processing (for a review see^7^), according to which the right hemisphere mainly processes negative emotions while the left hemisphere is involved in regulating positive emotions (*Valence Model*, Ehrlichman, 1986).

In light of these lateralized emotion processing models, previous research investigating the effect of sleep deprivation on emotion processing has yielded contradictory results. Some have found that sleep deprivation modulates brain response only to aversive stimuli^8–10^ which would indirectly support the Right Hemisphere Model. On the other hand, sleep deprivation has also been reported to be associated with enhanced reactivity toward reward-relevant, pleasure-evoking stimuli^4^ which are in line with the Valence Model. However, there seems to be some agreement, that REM sleep plays a specific role in modulating affective brain functions^11^, and there is evidence, that REM deprivation enhances lateralized brain activity as well^12,13^.

Domestic dogs (*Canis familiaris*) are widely used as model systems of socio-cognitive behavioural phenomena, due to their domestication history and the shared environment with humans^14–16^. Dogs’ often cited human-like skills include emotion processing, whereby they are able to respond to human emotional communicative cues^17^, discriminate emotional human faces from blank expressions^18^ or use human emotions as a discriminative cue in a learning task^18,19^.

There is a growing body of evidence that the emotional processing of dogs is also associated with hemispheric lateralization. Similarly to humans, dogs show left gaze-bias when looking at human faces, therefore processing them with the contralateral (right) hemisphere^20^. It is also suggested^21^ that dogs show lateralized behaviour in response to emotionally valenced cues. For example, seeing the owner amplifies right tail wagging (reflecting activation of the left hemisphere), which is associated with approach behaviour, while seeing a dominant unfamiliar dog amplifies left tail wagging (reflecting activation of the right hemisphere), which is associated with withdrawal behaviour^21,22^. Furthermore, dogs spontaneously recognize these lateralized tail wagging behaviours of other dogs and show concordant physiological, as well as behavioural responses^23^. Research on emotion recognition demonstrated, that while in the case of 4-year old children the gaze bias towards emotional facial expressions can be described by the Right Hemisphere Model, domestic dogs’ gaze pattern better fits the Valence Model^24^. 4-year-old children tend to show a left gaze bias toward both positive and negative emotional facial expressions, while domestic dogs tend to show a left gaze bias toward negative and a right gaze bias toward positive emotional faces^24^.

Dogs have also been proved to be excellent models for non-invasive comparative neuroscience^25^, including sleep-related neuro-cognitive measures (for a review see^26^). Recently, a non-invasive polysomnography method was adapted to family dogs^27^, and was used to test the effect of pre-sleep emotional experiences on sleep macrostructure^28^. The emotionally loaded social interactions affected subsequent sleep latency and caused a marked re-distribution of sleep stages. However, contrary to human findings, in dogs, negative emotional experience was associated with increased REM duration and shortened sleep latency^28^. Although these findings reveal intriguing differences and similarities between dogs’ and humans’ emotion processing during sleep, the investigation of the effect of sleep deprivation on emotion recognition in dogs is still missing from the literature.

In the present study, we therefore investigated the functional link between REM versus non-REM deprivation, and post-sleep emotion recognition among family dogs using an audio-visual matching task with human facial expression and non-verbal vocalizations of different valence. We hypothesized that similarly to humans^3,5,8,13,29^, disturbed sleep deteriorates performance in an emotion recognition task. Furthermore, we expected to find lateralized gazing behaviour both in response to the emotional stimuli per se (based on 25) as well as the lateralization-specific effect of sleep deprivation (based on 13,14).

## Materials and Methods

### Ethics statement

Research was carried out in accordance with the Hungarian regulations on animal experimentation and the Guidelines for the use of animals in research described by the Association for the Study Animal Behavior (ASAB). The Hungarian “Animal Experiments Scientific and Ethical Committee” issued a statement (under the number PE/EA/853-2/2016), approving our experimental protocol by categorizing it as a non-invasive study that causes less pain or suffering than the equivalent of inserting a needle. All owners volunteered to participate in the study and they gave written informed consent. Owners got a shopping voucher (of 5000 HUF value, approx. 15 EUR) in exchange for participating.

### Subjects

Task naïve adult pet dogs (N = 16) and their owners were recruited on a voluntary basis from a database at the Research Institute for Psychology. Participating in the sleep EEG research did not require prior training. One subject was excluded from the analysis because the owner withdrew from the experiment after the dog not being able to fall asleep for half-an-hour. The final sample consisted of 15 adult family dogs (2.57 (±0.71) years old; 6 males; from 11 breeds and 4 mongrels).

### General procedure

**Figure 1:**
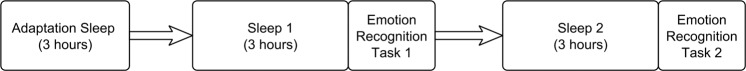
General procedure.

Subjects participated in polysomnography recordings of 3 h duration on a total of three occasions (see^27^ for detailed protocols) with approximately one week intervals between occasions (4–14 days). The first occasion was an adaptation session and thus was not analysed in the current study, but merely aimed to familiarize the subjects with the laboratory, the experimenter (E) and the electrode placement and to avoid a phenomenon known as the first-night effect in human literature^30^. The second and third occasions were the test occasions when dogs participated in the REM deprivation and the Sleep Interruption (non-REM deprivation) conditions in a counterbalanced order. The first 8 subjects started with REM deprivation condition (7 out of 8 included in the final sample) and the second 8 subjects started with Sleep interruption condition (all 8 dogs included in the final sample). Dog owners were always unaware of the type of sleep interruption. Both test occasions were followed by an emotion recognition task based on^31^. Figure 1 presents the general procedure.

### Sleep polysomnography measures

The recordings were always scheduled for the afternoon (starting time varied across dogs from 1 pm to 5 pm), because apart from the night time, dogs, similarly to humans, show the highest propensity to sleep during the afternoon^32^.

After a 5–10 minutes free exploration the owners settled on the mattress with their dogs and helped the research staff by gently holding the dogs’ head while the surface electrodes were being placed on the dogs’ head and body. Dogs’ fur does not need to be shaved. Electrode placement can be done in the same way as with human hair independently of fur type. During electrode placement, all dogs were reinforced using social reinforcement (e.g., petting, praise) and/or food reward. Two electrodes were placed on the right and left zygomatic arch next to the eyes (F7, F8), and another two over the anteroposterior midline of the skull (Fz, Cz). All four EEG electrodes were referred to the G2 electrode which was in the posterior midline of the skull (occiput; external occipital protuberance). The ground electrode (G1) was attached to the left musculus temporalis. To detect the dogs’ heart-rate ECG electrodes were placed bilaterally over the second rib. Gold-coated Ag|AgCl cup electrodes fixed with EC2 Grass Electrode Cream (Grass Technologies, USA) were used. Impedances for the EEG electrodes were kept below 20 kΩ.

The signal was collected, pre-filtered, amplified, and digitized with a sampling rate of 1024 Hz/channel using a SAM 25 R style MicroMed Headbox (MicroMed Inc., Houston, TX, USA). The hardware passband was set at 0.5–256 Hz, sampling rate of 512 Hz, anti-aliasing filter with cut-off frequency at 1 kHz, and 12-bit resolution covering a voltage range of ±2 mV as well as second-order software filters (high pass >0.016 Hz, low pass <70 Hz) using System Plus Evolution software (MicroMed Inc, Houston, TX, USA).

Before arriving at the laboratory owners were informed about the estimated time of the test (3.5 hours). All dogs were house-trained. The sleep laboratory was equipped as an ordinary room with a mattress on the floor, three small pillows and a blanket. Owners could decide whether they preferred their dog to sleep on the mattress with them or on the floor next to them. There was no window in the room and lights were always turned of providing constant dark in the room. After a 5–10 minutes exploration and familiarization the owner took place on the mattress and assisted E throughout the process of fixing surface attached electrodes onto the dog. The dog was rewarded with food during electrode placement if the owner deemed it necessary; social reinforcement (praise, petting) was used in all cases. After the electrode placement was done the E left the room and switched off the light providing constant darkness in the room. While the dog was resting or sleeping, the owner could watch a movie with an earphone or sleep as well. Owners asked to stay quiet and still on the mattress and not to wake up the dog. Furthermore, owners are asked not to leave the room during the polysomnography recording. E monitored the recording from the adjacent room. In the REM Deprivation condition (RD), E woke up the dog by entering the sleeping room, when she recognized the first signs of REM sleep. These signs could be rapid eye movements, fast EEG activity, muscular atonia, and irregular heart beat (c.f. 28). After E entered the room, she talked to or petted the dog for 2 minutes in order to keep the subject awake and avoid for it to relapse immediately into REM sleep again^33^.

In the Sleep Interruption condition (SI) there were 2 potential scenarios, depending on if the dog started with RD or SI condition. If the first occasion was RD, subjects (N = 7) in the SI condition were woken up at the exact same time points from sleep onset as during RD; unless they were in a REM phase, in which case the awakening occurred at the first non-REM or drowsiness after REM. Dogs were kept awake for 2 minutes in the same way as in RD condition. For dogs whose first occasion was SI condition (N = 8), the awakenings from non-REM or drowsiness started after 2 REM phases to ensure that they will have REM sleep. An attempt was made to equalize the number of awakenings with the RD condition, so an average 6 times of awakenings were carried out.

Polysomnography recordings of dog sleep were coded with a self-developed software (Fercio © Ferenc Gombos, 2012) according to standard criteria^27^. Wakefulness, drowsiness, non-REM and REM sleep were coded in 20 s epochs by inspecting the EEG, EOG and ECG channels. The rating was always blind to subject details and conditions. The following 7 variables were exported from the hypnograms: sleep efficiency (time spent asleep relative to the total length of the recording, %), relative wake duration (time spent awake relative to the total length of the recording, %), WASO (waking after sleep onset after first drowsiness and after first Non-REM sleep, min), sleep latency (until first drowsiness and until first non-REM sleep, min), relative drowsiness duration (%), relative non-REM duration (%) and relative REM duration (%). Descriptive statistics of the conditions are presented in Table 1. Data were normally distributed. Additionally, the number of awakenings the experimenter performed were noted and compared between the two conditions.

**Table 1:** Descriptive statistics on sleep macrostructure differences between REM Deprivation and Sleep Interruption conditions.

		Mean (SD)	Minimum	Maximum
Sleep efficiency (%)	REM Deprivation	51.88 (15.87)	22.61	79.78
	Sleep Interruption	50.09 (18.66)	18.40	88.42
Relative wake duration (%)	REM Deprivation	48.09 (15.88)	20.22	77.39
	Sleep Interruption	49.59 (18.71)	11.58	80.69
WASO after first drowsiness (min)	REM Deprivation	75.98 (23.37)	34.00	112.67
	Sleep Interruption	78.00 (33.64)	20.00	139.67
WASO after first S2 sleep (min)	REM Deprivation	71.20 (21.78)	34.00	112.67
	Sleep Interruption	76.13 (32.28)	19.00	130.00
Sleep latency after first drowsiness (min)	REM Deprivation	11.38 (8.73)	2.00	27.67
	Sleep Interruption	12.06 (10.79)	1.00	37.33
Sleep latency after first S2 sleep (min)	REM Deprivation	21.78 (11.92)	6.67	46.00
	Sleep Interruption	19.02 (10.62)	6.67	40.00
Relative drowsiness duration (%)	REM Deprivation	45.27 (15.04)	27.48	82.55
	Sleep Interruption	39.30 (20.69)	7.90	99.01
Relative Non-REM duration (%)	REM Deprivation	46.82 (13.46)	16.11	61.85
	Sleep Interruption	31.01 (12.37)	0.99	53.57
Relative REM duration (%)	REM Deprivation	7.91 (5.36)	0.00	15.24
	Sleep Interruption	29.69 (12.85)	0.00	49.27

### Emotion recognition task

**Figure 2:**
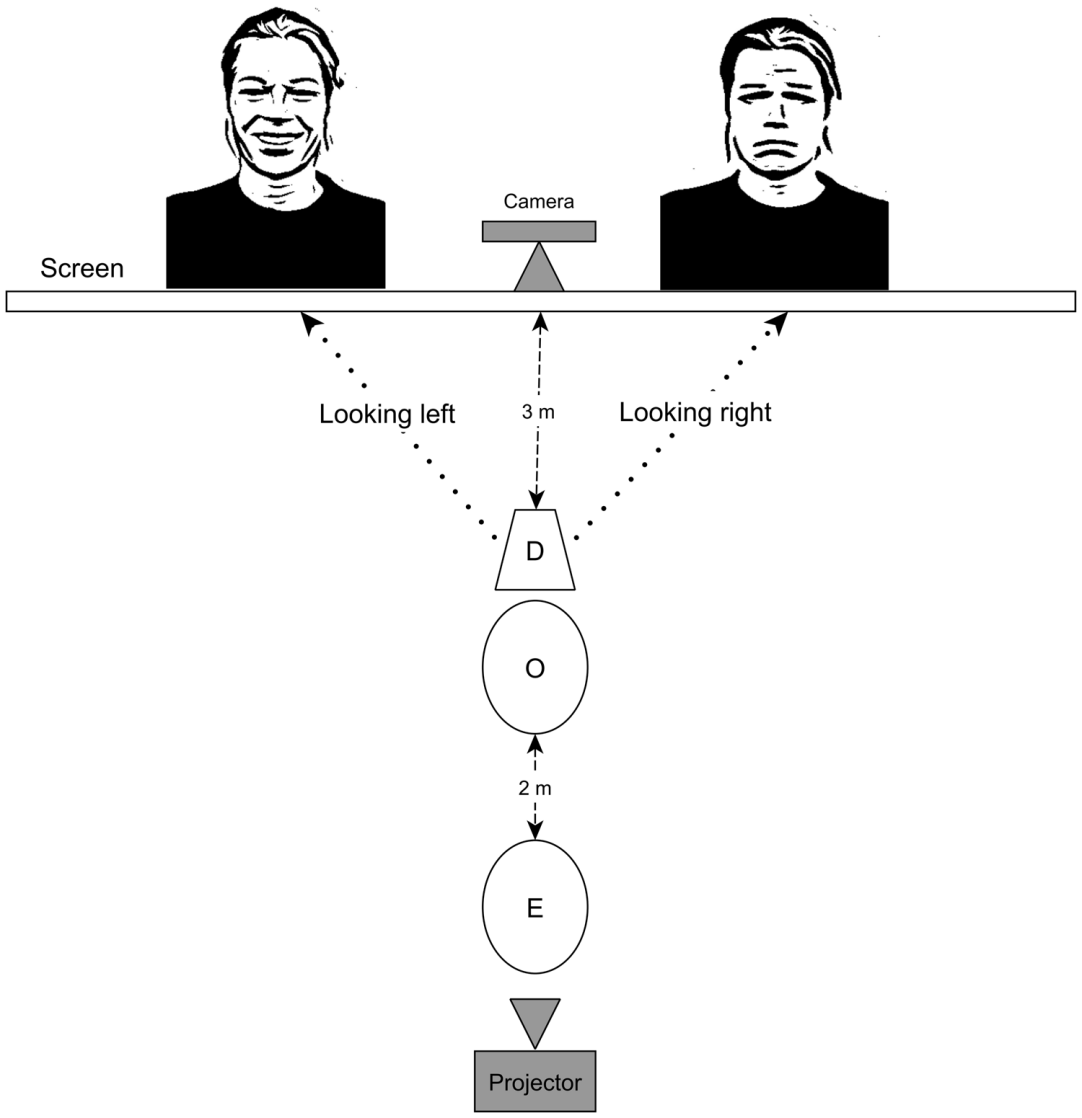
Experimental setup for the emotion recognition task (happy face left, sad face right). E = Experimenter, O = Owner, D = Dog.

Following the 3 hours sleep, the electrodes were taken off from the dogs’ scalp. Dogs were allowed to drink. The E, the Owner (O), and the Dog (D) entered a different test room where the emotion recognition task was carried out. E asked O to sit down to the chair in front of the screen and hold D by the collar during the test session between his/her legs. E informed O that 2 test sessions would be conducted with a 3 minutes break and each session would consist of 10 pairs of pictures and a sound. E was standing 3 meters behind the chair, facing the screen as well. Figure 2 presents the experimental setup of the emotion recognition task.

Each trial started with presenting an attention grabber for 5 sec in the middle of the screen. Subjects were then presented simultaneously with a pair of emotional face images as well as an emotional sound playback. Images were taken from the Radboud Faces Database^34,35^, and for each given trial the two images, projected to the left and right respectively, were depicting the same individual but with different expressions (positive: happy vs. negative: sad). The sound playback was a single human vocalization of either positive (laugh) or negative (cry) valence from the same individual (database: 35) or a neutral sound (brownian noise) following the procedure by^31^. A trial consisted of the presentation of a combination of the acoustic and visual stimuli and lasted 5 s. The order of stimuli was counterbalanced regarding Voice and side of the congruent picture. Stimuli appeared in different order for each dog and each test session. The 2 × 10 trials presented different stimulus combinations. 16 face-pairs (4 women, 4 men after the first sleep occasion and 4 different women, 4 different men after the second sleep occasion) × 2 vocalizations for both sleep occasions (laughing or crying) × 2 face positions (left or right), in addition to 4 control trials (4 face-pairs with neutral noise). Face-pairs and vocalizations were presented in a counterbalanced order. The same face-pair or same vocalization was presented maximum twice right after each other. Therefore, each subject saw each possible combination once (see Supplementary 1).

Dogs were presented with a total of 20 trials with a 3 minutes break after the first 10 trials. A video camera recorded the subjects’ spontaneous looking behaviour. Looking behaviour was coded frame by frame with Solomon Coder in 0.2 sec resolution (beta 091110, ©2006e 2008 by András Péter, http://solomoncoder.com/). For each frame dogs’ behaviour was categorized as looking right, looking left, or looking elsewhere. The duration of looking right or left was exported (in seconds) for each of the 20 trials for all dogs. Trials in which dogs did not look at the picture at all were excluded from the analysis. The average time dogs spent looking at the pictures was 3.1 ± 1.8 sec with a minimum of 0.4 sec looking time/trial. Dogs successfully completed on average 42.5% ± 16.03 of trials.

### Statistical analysis

The REM deprivation (RD) and Sleep interruption (SI) conditions were compared using paired sample t-tests with regards to all exported sleep macrostructure variables (Table 1). Data were normally distributed.

Generalized Linear Mixed Models were performed to analyse the total time (sec) spent looking left or right during the emotion recognition task. As the RD and SI conditions differed in three macrostructure variables by the paired t-test (see Results), while such macrostructure variables are inherently interrelated, separate models were built for each. Side (the place of the voice-congruent picture; left or right) and voice (sad, happy or neutral) were entered in all models with one sleep macrostructure variable (REM sleep duration, Non-REM duration, number of awakenings by the experimenter) as fixed factors. Thus a total of 3 GLMMs were run for looking left and another 3 for looking right. ID was included as a random factor. All possible two-way and three-way interactions of the fixed factors were tested. Statistical tests were two-tailed, the α value was set at 0.05. All statistical analyses were carried out using SPSS software package (v. 21.0).

## Results

Paired sample t-tests were conducted to compare the SI and RD conditions regarding sleep macrostructure (see Table 1). As expected there was more REM sleep in the SI condition (t_14_ = −6.74, p < 0.001) and in turn, there was more Non-REM sleep in the RD condition(t_14_ = 4.71, p < 0.001). There were no significant differences between the two conditions in any other sleep structure variables (Sleep efficiency, Relative wake duration, WASO after first drowsiness, WASO after first S2 sleep, Sleep latency after first drowsiness, Sleep latency after first S2 sleep, and Relative drowsiness duration; all p > 0.05). There was, however, a significant difference in the number of awakenings (t_14_ = 3.68, p = 0.002, M_RD_ = 6.93, SD_RD_ = 3.41; M_SI_ = 4.93, SD_SI_ = 2.63) with more awakenings by the experimenter in the RD condition. Because the conditions differed in three variables (Non-REM sleep, REM sleep, and the number of awakenings), we analysed the looking behaviour in the emotion recognition task along with these variables in different models.

**Figure 3:**
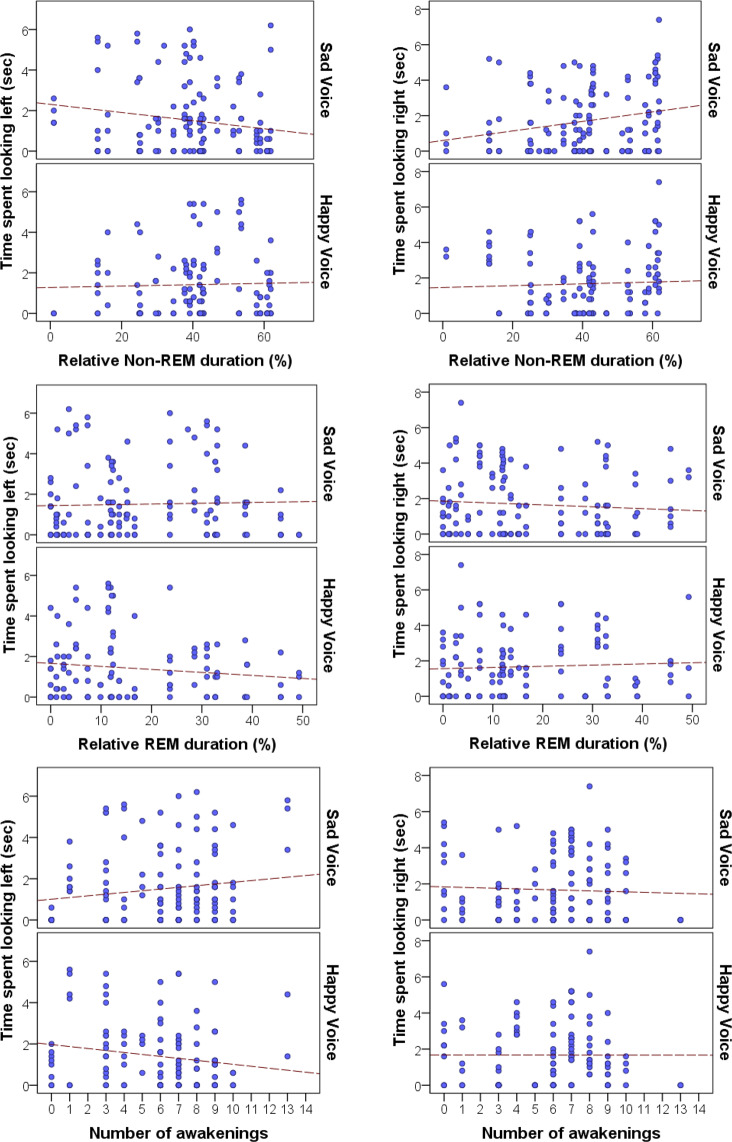
The associations between looking behaviour (left or right) and sleep macrostructure variables.

Regarding the *time spent looking right* (T_right_) three models were tested with one macrostructural variable in each (REM duration, Non-REM duration, number of awakenings) plus Voice and Side as fixed factors. Relative Non-REM duration was significantly (positively) associated with the time spent looking right (F_1,246_ = 5.794, p = 0.017), but neither the emotional content of the voice (Voice; F_1,247_ = 1.906, p = 0.169) nor the place of the voice-congruent image (Side; F_1,246_ = 0.692, p = 0.406) had an effect and all interactions were non-significant (p > 0.05). Relative REM duration had no significant effect (F_1,246_ = 0.507, p = 0.477), and again neither Voice (F_1,246_ = 0.890, p = 0.346) nor Side (F_1,246_ = 0.002, p = 0.966) had an effect on the time that dogs spent looking at the facial image on the right side. The number of awakenings had a significant effect (F_1,246_ = 2.251, p = 0.016) with slightly more time spent looking right with an increasing number of awakenings. Voice (F_1,246_ = 0.012, p = 0.913) and Side (F_1,246_ = 1.004, p = 0.318) had no significant effect and all interactions were non-significant (p > 0.05, Fig. 3).

Regarding the *time spent looking left* (T_left_) relative Non-REM duration (F_1,246_ = 0.544, p = 0.462) and Side (F_1,246_ = 1.374, p = 0.242) had no effect on time spent looking left. But the emotional content of the voice had a significant effect (Voice; F_1,246_ = 4.073, p = 0.045), as dogs spent marginally more time looking at the picture presented on the left when there was a sad playback sound. Relative REM duration also had no effect (F_1,246_ = 0.863, p = 0.354), and in this model neither Voice (F_1,246_ = 0.337, p = 0.562) nor Side (F_1,246_ = 0.105, p = 0.747) influenced looking to the left. The model that included the number of awakenings as fixed factor failed to show significant main effects of fixed factors (all p values > 0.05). But the number of awakenings was in interaction with Voice (F_1,246_ = 5.416, p = 0.021), an increased number of awakenings was related to looking more to the left when there was a sad sound playback while looking less to the left when there was a happy sound playback (Fig. 3).

## Discussion

Following up on previous research suggesting that among humans, sleep deprivation deteriorates performance in emotional tasks^3,5,13^ in the present study we provide evidence that in family dogs selective deprivation of REM versus non-REM sleep affects behavioural performance in an emotion processing task.

Sleep deprivation studies, combined with invasive methodology (cisternal puncture/cerebrospinal fluid extraction), were conducted on dogs more than a century ago^36^, however, to our best knowledge, the present study is the first in which a sleep stage specific deprivation paradigm was successfully adapted to family dogs and carried out with a fully non-invasive methodology. As intended, subjects in the REM Deprivation (RD) condition spent significantly less time in REM sleep and more in non-REM sleep when compared with the Sleep Interruption (SI) condition, while sleep efficiency and other key sleep macrostructure variables were the same between these conditions. However, our study has a methodological limitation; despite our attempt to equalize the number of awakenings by the experimenter, there were significantly more awakenings in the RD condition. This potential problem was handled in the present study by building separate models for the different macrostructural variables. However, future research might consider to apply less strict rules, e.g. on the presence of REM sleep in the SI condition (our criteria was to have two full REM phases before any interruption could take place) and/or to use a between-subject design (at the expense of having to recruit considerably more subjects), where matched pairs of subjects can be awakened at the exact same time points using data from the RD condition.

Regarding the emotion recognition task, in contrast to^31^, dogs in our study did not show evidence of cross-modal integration of heterospecific (human) emotional stimuli (in which case the happy/sad valence of Voice was expected to have an interactive effect with left/right voice-congruent Side of the visual stimuli). One potential reason for such discrepancy between the two studies is that in contrast to^31^, in the present study dogs in both the RD and SI conditions experienced disturbed sleep immediately before the emotion recognition task. It is expected that any kind of sleep disturbance would damage the subjects’ capacity to integrate cross-modal emotional information (see e.g. human literature about sleep deprivation’s effect on vigilance and other cognitive domains which are known to influence performance in affective tasks as well^37^).

Our results are in line with another body of previous research regarding dogs’ emotional processing^24^, whereas dogs are known to show a left gaze bias upon being presented with negatively valenced or neutral (but not positive) emotional stimuli, in the former case facial expressions, in the present study sad voice playback. The present study thus gives further empirical evidence to the Valence Model^38^ of the emotion process in dogs, suggesting right hemisphere lateralization for processing negative emotions. Although it has to be noted that previous studies^24^ that contrasted the time spent looking left to chance level (in cases when either negative, neutral, or positive emotional stimuli were perceived) found a left gaze bias towards both negative and neutral expressions, whereas no significant bias was found towards positive expressions. In the present study, we contrasted negative and positive acoustic emotional stimuli and found a left bias in case of negative emotional stimuli. It seems, therefore, that the left bias is more pronounced in case of acoustic emotional stimuli compared to visual. Moreover, our findings parallel to those of others which suggest that sleep deprivation modulates brain responses mainly to aversive stimuli^8–10^.

Regarding the effect of ‘sleep stage specific’ deprivation on the looking behaviour in the emotion recognition task, we found that non-REM duration was positively associated with right gaze bias, suggesting left-hemisphere dominance. This finding is contradictory to human lateralized brain functions, suggesting that sleep deprivation produces lateralized deficits in visual attention particularly in the left visual field^5^. Others, however, argue against this hypothesis and demonstrate a global and bilateral performance deficit in attention^13^. In addition to the controversies in the human sleep deprivation literature alone, there are several procedural differences between those and the present study which can potentially influence the research outcome, including the complexity of the emotional stimuli and the fact that an interspecific context was used. It has also been shown in an fMRI study^39,40^ that speech intonation processing is differently lateralized in the dog brain than in the human brain.

The interactional effect of the number of awakenings with voice valence on the time spent looking left suggests a dominance of the right hemisphere. Being woken up is in itself a potential stressor for the sleeping animal, therefore more frequent awakenings can be translated to increased stress, explaining the higher sensitivity to negative stimuli (i.e. sad voice) and more dominant activation of the right hemisphere, supporting the Valence Model of emotion processing^38^.

In summary, this paper presents results of a pioneering research on the effects of disturbed sleep on interspecific emotion recognition in dogs. The link between sleep and emotions has been in the focus of research from decades, but results are still contradictory, and little is known from a comparative aspect^1,29^. Our findings suggest that sleep stage specific deprivation has a selective effect on dogs’ performance in an emotion recognition task.

## Data Availability

All data used in these analyses are available as electronic supplementary material.
